# Soluble tumor necrosis factor-alpha-induced hyperexcitability contributes to retinal ganglion cell apoptosis by enhancing Nav1.6 in experimental glaucoma

**DOI:** 10.1186/s12974-021-02236-6

**Published:** 2021-08-21

**Authors:** Shuo Cheng, Hong-Ning Wang, Lin-Jie Xu, Fang Li, Yanying Miao, Bo Lei, Xinghuai Sun, Zhongfeng Wang

**Affiliations:** 1 State Key Laboratory of Medical Neurobiology and MOE Frontiers Center for Brain Science, Institutes of Brain Science, Fudan University, Shanghai, 200032 China; 2 Institute of Neuroscience and Third Affiliated Hospital, Henan Provincial People’s Hospital, Henan Eye Institute, Henan Eye Hospital, People’s Hospital of Zhengzhou University, Zhengzhou University, Zhengzhou, 450003 China; 3 Department of Ophthalmology at Eye & ENT Hospital, Shanghai Key Laboratory of Visual Impairment and Restoration, Fudan University, Shanghai, 200031 China

**Keywords:** TNF-α, Nav1.6, Hyperexcitability, Neuroinflammation, Retinal ganglion cells, Apoptosis, Glaucoma

## Abstract

**Background:**

Neuroinflammation plays an important role in the pathogenesis of glaucoma. Tumor necrosis factor-alpha (TNF-α) is a major pro-inflammatory cytokine released from activated retinal glial cells in glaucoma. Here, we investigated how TNF-α induces retinal ganglion cell (RGC) hyperexcitability and injury.

**Methods:**

Whole-cell patch-clamp techniques were performed to explore changes in spontaneous firing and evoked action potentials, and Na^+^ currents in RGCs. Both intravitreal injection of TNF-α and chronic ocular hypertension (COH) models were used. Western blotting, immunofluorescence, quantitative real-time polymerase chain reaction (q-PCR), and terminal deoxynucleotidyl transferase dUTP nick end labeling (TUNEL) techniques were employed to investigate the molecular mechanisms of TNF-α effects on RGCs.

**Results:**

Intravitreal injection of soluble TNF-α significantly increased the spontaneous firing frequencies of RGCs in retinal slices. When the synaptic transmissions were blocked, more than 90% of RGCs still showed spontaneous firing; both the percentage of cells and firing frequency were higher than the controls. Furthermore, the frequency of evoked action potentials was also higher than the controls. Co-injection of the TNF-α receptor 1 (TNFR1) inhibitor R7050 eliminated the TNF-α-induced effects, suggesting that TNF-α may directly act on RGCs to induce cell hyperexcitability through activating TNFR1. In RGCs acutely isolated from TNF-α-injected retinas, Na^+^ current densities were upregulated. Perfusing TNF-α in RGCs of normal rats mimicked this effect, and the activation curve of Na^+^ currents shifted toward hyperpolarization direction, which was mediated through p38 MAPK and STAT3 signaling pathways. Further analysis revealed that TNF-α selectively upregulated Nav1.6 subtype of Na^+^ currents in RGCs. Similar to observations in retinas of rats with COH, intravitreal injection of TNF-α upregulated the expression of Nav1.6 proteins in both total cell and membrane components, which was reversed by the NF-κB inhibitor BAY 11-7082. Inhibition of TNFR1 blocked TNF-α-induced RGC apoptosis.

**Conclusions:**

TNF-α/TNFR1 signaling induces RGC hyperexcitability by selectively upregulating Nav1.6 Na^+^ channels, thus contributing to RGC apoptosis in glaucoma.

## Introduction

Glaucoma, the leading cause of irreversible blindness, is a retinal neurodegenerative disease, which affects over 60 million people around the world [1–3]. Progressive apoptotic death of retinal ganglion cells (RGCs) and degeneration of RGC axons and dendrites, resulting in visual field loss, are the fundamental pathogenesis of glaucoma [4–8]. Although aging is the chief risk factor for the development of glaucoma and sensitivity of ocular tissues to elevated IOP is an associated risk factor [9, 10]; however, reduction of IOP could not completely prevent the pathological progression of glaucoma [11, 12], suggesting that the mechanisms underlying RGC loss in glaucoma are complicated. Increasing evidence indicates that neuroinflammation induced by excessive pro-inflammatory factors, which are released from activated retinal glial cells, plays significant roles in RGC damage in glaucoma [13–15].

Tumor necrosis factor-alpha (TNF-α), a classical inflammation cytokine, exerts multiple functions in the nervous system by binding to two types of TNF-α receptor (TNFR1 and TNFR2) [16, 17]. Soluble TNF-α (17 kDa) preferentially binds to TNFR1, leading to neuroinflammation and cell death [18–20], while transmembrane TNF-α (22 kDa) primarily binds to TNFR2 that mediates neuroprotective effects [21, 22]. TNF-α also plays important roles in the pathogenesis of retinal diseases, such as glaucoma. Previous studies have demonstrated that TNF-α was involved in retinal axon loss and RGC death in glaucoma [23–25]. TNF-α-induced RGC death in glaucoma could be mediated by multiple pathways. TNF-α caused RGC loss by activation of death signaling, such as caspase 8 and oxidative stress [26]. In chronic ocular hypertension (COH) model, soluble TNF-α induced GluA2 subunit of AMPA receptor endocytosis and activated Ca^2+^-permeable GluA2-lacking AMPA receptors in RGCs, thus promoting RGC death [24]. Direct neutralization of soluble TNF-α in the retina of experimental glaucoma was able to reduce RGC death efficiently [24, 27].

In a previous study, we have shown that in a rat experimental glaucoma model, IOP elevation led to depolarized resting membrane potential in RGCs, and the cells displayed hyperexcitability, which was characterized by increased spontaneous firing. The hyperexcitability of RGCs could be attenuated by intravitreal pre-injecting the TNF-α antagonist XPro1595 and the nitric oxide (NO) antagonist L-NAME, indicating that inflammatory factors released from retinal glial cells may be involved [28]. Neuronal hyperexcitability has been demonstrated to be associated with cell apoptosis [29–31]. Changes in ion channels may contribute to the production of hyperexcitability, especially voltage-gated K^+^ and Na^+^ channels. Although it is reported that TNF-α could modulate outward K^+^ currents in RGCs [32], the mechanisms underlying TNF-α-induced hyperexcitability of RGCs in glaucoma are largely unknown. In this study, we show that TNF-α enhanced RGC excitability by upregulating Nav1.6 channels through activating TNFR1, thus contributing to RGC apoptosis.

## Materials and methods

### Animals

Male Sprague-Dawley rats (4 weeks old, weighing 100~110 g) were obtained from the SLAC Laboratory Animal Co., Ltd. (Shanghai, China) and housed under a 12-h light/dark cycle with enough food and water. All animal experiments were performed in accordance with the National Institutes of Health (NIH) guidelines for the Care and Use of Laboratory Animals and were approved by the Institutes of Brain Science at Fudan University. All the experiments described in this study were performed by researchers that were blind with respect to the treatments.

### Intravitreal injection

Recombinant rat TNF-α (5 ng in 1 ml of 0.9% saline with 0.1% BSA, R&D systems, Minneapolis, MN, USA) (2 μl) was injected into the right vitreous cavity of the anesthetized rat with a micro-injector (Hamilton, Reno, NV, USA) under a stereoscopic microscope (Carl Zeiss). Given that the vitreous volume is ∼20 μl [33, 34], the concentration of TNF-α in the vitreous cavity is ∼0.5 ng/ml. 2 μl of 8-chloro-4-(phenylthio)-1-(trifluoromethyl)-[1,2,4]triazolo[4,3-*a*]quinoxaline (R7050, 10 μM, Tocris Bioscience, Ellisville, USA), BAY 11-7082 (10 μM), stattic (10 μM), or SB203580 (10 μM) (Selleck Chemical, Houston, TX) was intravitreally injected in the same manner. The eyes that received saline injection were used as controls. Details of the operation were described in our previous reports [28, 35].

### Rat COH model

Rat COH model was produced in accordance with previous studies [28, 35]. In brief, the rat was anesthetized with 10% chloral hydrate (4 ml/kg, i.p.), and the operation eye (right eye) was further anesthetized with 0.4% oxybuprocaine hydrochloride eyedrop (Benoxil, Santen Pharmaceutical Co., Ltd., Osaka, Japan). The micro-magnetic beads (10 μl, diameter ≈ 10 μM, BioMag® Superparamagnetic Iron Oxide, Bangs Laboratories, Ins) were slowly injected into the anterior chamber under an OPMI VISU 140 microscope (Carl Zeiss, Jena, Germany). A small handheld magnet (0.45 Tesla) was used to distribute the microbeads evenly around the iridocorneal angle. The IOP was measured using a handheld digital tonometer (Tonolab, TioLat, Finland) under general anesthesia as described above. The average value of five consecutive measurements with a deviation of < 5% was accepted. The IOPs of both eyes were measured before surgery (baseline), immediately after operation (day 0), the day after the operation (day 1, COH1D), the third day (COH3D), and 1 and 2 weeks after the operation (COH1W, COH2W). All the measurements were performed at 9:00–10:00 a.m. to avoid the possible influence of circadian rhythms on IOP [36].

### Preparation of retinal slices and isolated RGCs

Rat retinal slices (200 μm in thickness) were made on a Leica Vibrotome (VT1000S) and incubated in carbogen saturated (95% O_2_ and 5% CO_2_) artificial cerebral spinal fluid (ACSF) containing the following (in mM): 125 NaCl, 2.5 KCl, 25 NaHCO_3_, 1.25 NaH_2_PO_4_, 2.5 CaCl_2_, 1 MgCl_2_, and glucose 10 (pH 7.4) for 30 min at room temperature (22–24°C) before recording. To isolate RGCs, the retinas of anesthetized rats were removed quickly and incubated in the oxygenated Hank’s solution containing the following (in mM): NaCl 137, NaHCO_3_ 0.5, NaH_2_PO_4_ 1, KCl 3, CaCl_2_ 2, MgSO_4_ 1, HEPES 20, and glucose 16 (pH 7.4). After digesting in Hank’s solution with 1.6 U/ml papain (Worthington Biochemical, Freehold, NJ, USA) and 0.2 mg/ml L-cysteine for 26 min at 34°C, retinal neurons were obtained by mechanical dissociation. The detailed procedures were described in our previous reports [37, 38].

### Patch-clamp recordings

Whole-cell patch-clamp experiments were performed using a patch-clamp amplifier (Axopatch 700B) and Digidata 1440A (Molecular Devices, Foster City, CA, USA) at room temperature. Patch pipettes (BF150-86-10 glass, Sutter Instrument Co., Novato, CA, USA) were pulled by a P-97 Flaming/Brown micropipette puller (Sutter Instrument) with a resistance of 5–8 MΩ after fire-polishing (Model MF-830, Narishige, Japan).

For spontaneous firing recordings in RGCs, the individual retinal slice was continuously superfused with oxygenated ACSF at a rate of 1–2 ml/min at room temperature. RGCs in retinal slices were identified with the help of infrared-differential interference contrast (IR-DIC) video microscopy (Olympus, Japan) as described [28, 38]. Patch pipette solution contained (in mM): 120 potassium d-gluconate, 1 EGTA, 10 HEPES, 4 ATP-Mg, 0.3 GTP-Na, 10 phosphocreatine, 0.1 CaCl_2_, 1 MgCl_2_, and Alexa Fluor 488 (pH 7.2 adjusted with KOH, 290–300 mOsm/L). To record Na^+^ currents, the bath solution was consisted of (in mM) 130 NaCl, 2 CaCl_2_, 1 MgCl_2_, 10 HEPES, 15 tetraethylammonium (TEA)-Cl, 10 4-aminopyridine (4-AP), and 10 glucose (pH 7.4 with NaOH, 300–310 mOsm/L with sucrose). The patch pipette solution contained (in mM) 130 CsCl, 10 NaCl, 5 HEPES, 8 EGTA, 10 TEA-Cl, 2 ATP-Mg, and 1 GTP-Na (pH 7.2 adjusted with CsOH, 290–300 mOsm/L with sucrose). Na^+^ currents were induced by a series of 50 ms depolarizing voltage pulses from a holding potential of −70 to +30 mV in increments of 10 mV. The inactivation curves of voltage-gated sodium channels (VGSCs) were detected by giving RGC a 200 ms pre-pulse from −70 mV (holding potential) to different voltages and then depolarizing to −10 mV. Both activation and inactivation curves were fitted by Boltzmann function.

### Western blotting

Western blotting analysis was conducted following the procedures previously described [35]. Briefly, retinal total proteins or membrane proteins were extracted, and protein concentrations were determined with a bicinchoninic acid (BCA) assay kit (Pierce Biotechnology, IL, USA). Protein samples were then separated by SDS-PAGE gel in 6 or 10% and transferred to a PVDF membrane (Immobilon-P, Millipore, Billerica, MA, USA). The PVDF membrane was blocked for 6 h in 5% non-fat powdered milk and then incubated with primary antibody overnight at 4°C. In this study, the following primary antibodies were used: monoclonal mouse anti-β-actin (1:3000 dilution, Sigma-Aldrich, St. Louis, MO, USA), anti-TNF Receptor 1 (1:5000 dilution, Abcam, Cambridge, MA, USA), anti-TNF Receptor 2 (1:12000 dilution, Abcam), anti-Nav1.6 (1:600 dilution, Alomone Labs, Israel), and anti-GAPDH (1:1000 dilution, Cell Signaling Technology, MA, USA). The membranes were then incubated at room temperature for 1 h with donkey anti-mouse, rabbit, or goat IgG HRP (Jackson ImmunoResearch Labs, Wes Grove, PA, USA). The blots were visualized by an Odyssey near-infrared imaging scanner (FluorChem E System, Protein Simple, USA).

### Immunohistochemistry

The protocol of immunofluorescence staining was followed as described in previous studies [35]. In short, dissected eyes were dehydrated with 10%, 20%, and 30% sucrose solutions gradually and fixed with 4% paraformaldehyde at 4°C for 12 h. Retinal slices at 14-μm thickness were vertically sectioned and blocked with a mixture of 3% bovine serum and PBS on chrome-alum-gelatin-coated slides (Thermo-Fisher Scientific, Pittsburgh, PA, USA). The slices were incubated with the primary antibodies: anti-NaV1.6 (1:600 dilution, Alomone Labs) and anti-Brn-3a (1:400 diluted, Santa Cruz Biotechnology, Santa Cruz, VA, USA) at 4°C for 48 h. For negative control, Nav1.6 antibody was pre-absorbed by Nav1.6 blocking peptide (Alomone Labs). Then, the slices were incubated with Cy3- or Alexa Fluor 488-labeled secondary antibodies (1:400 diluted, Sigma-Aldrich) for 2 h at room temperature. 150 μl of the DAPI working solution was added to each slide for detecting cell nuclei. After washing, the sections were covered with an anti-fade mounting medium and photographed with FluoView 1000 confocal microscope (Olympus, Tokyo, Japan).

### Terminal deoxynucleotidyl transferase dUTP nick end labeling (TUNEL)

To detect neuronal apoptosis, the deoxynucleotidyl transferase-mediated biotinylated UTP nick end labeling (TUNEL) assay was performed on whole flat-mounted retinas [35]. The DeadEnd Fluorometric TUNEL System G3250 kit (Promega, Madison, WI, USA) was used according to the manufacturer’s instructions. The whole retina was analyzed, and all TUNEL-positive signals that merged well with DAPI were counted. The fluorescence images were captured using the confocal microscope through a 20× objective (FluoView 1000, Olympus, Japan).

### Q***uantitative*** real-time PCR

Total RNA was extracted from the whole retina using the TaKaRa MiniBEST Universal RNA Extraction Kit (#9767, Takara, Japan). Q***uantitative*** real-time polymerase chain reaction (q-PCR) was carried out using the PrimeScript™ RT reagent Kit with gDNA Eraser (#RR047A, Takara, Japan), and mRNA was measured using a TB Green® Premix Ex Taq™ II (#RR820A, Takara, Japan). The qPCR assays were performed on the QuantStudio 3 Real-Time PCR system (Thermo Fisher Scientific, USA). The relative mRNA levels were normalized from Ct values according to 2^-△△ct^ calculation method. The sequences of primers used in this study are as follows: Nav1.1, forward 5′-GCG ATT ATG TGA CAA GCA TTT TG-3′, reverse 3′-CGG AGG GAG ATG AGC TTC AG-5′; Nav1.2, forward 5′-TTC ATG GCT TCC AAT CCC TCC-3′, reverse 3′-GGT GTC ACG TCA GTC TTC TCT-5′; Nav1.6, forward 5′-GCA AGC TCA AGA AAC CAC CC-3′, reverse 3′-CCG TAG ATG AAA GGC AAA CTC T-5′; β-actin, forward 5′-AGC CAT GTA CGT AGC CAT CC-3′, reverse 3′-CTC TCA GCT GTG GTG GTG AA-5′.

### Reagents and drugs

R7050, d-(-)-2-amino-5-phosphonopentanoic acid (D-APV), bicuculline, CNQX, 4,9-anhydrotetrodotoxin (AHTTX), and TTX were purchased from Tocris (Tocris Bioscience, Ellisville, MO, USA). BAY 11-7082, SB203580 and stattic were from Selleck (Selleck Chemical), and the others were from Sigma-Aldrich (St. Louis, MO, USA).

### Statistical analysis

Data were analyzed using GraphPad Prism (version 6.02, Graphpad Software Inc., USA), Clampfit 10.2 (Molecular Devices, Foster City, CA, USA), Igor 4.0 (WaveMetrics, Lake Oswego, OR, USA), and Origin 2018 (OriginLab, Northampton, MA, USA). In this work, the “*n*” represents cell number in electrophysiological experiments, or animal number in Western blotting, q-PCR, and TUNEL experiments. A Boltzmann function was used to fit the activation and inactivation curves. All experiments and measurements were performed in quadruplicate minimally and analyzed by *t* test, Brown-Mood test, Mann-Whitney test, or one-way ANOVA with Bonferroni’s post hoc test (multiple comparisons). Before performing *t* test or one-way ANOVA analysis, the data were analyzed using Shapiro-Wilk test or Brown-Forsythe test to evaluate the normality or the homogeneity of variance. Membrane potential and frequency of spontaneous firing were presented as a median and interquartile range, while the other data were expressed as mean ± SEM. The threshold for statistical significance was *P* < 0.05 in all tests.

## Results

### TNF-α/TNFR1 pathway contributes to RGC hyperexcitability

**Fig. 1:**
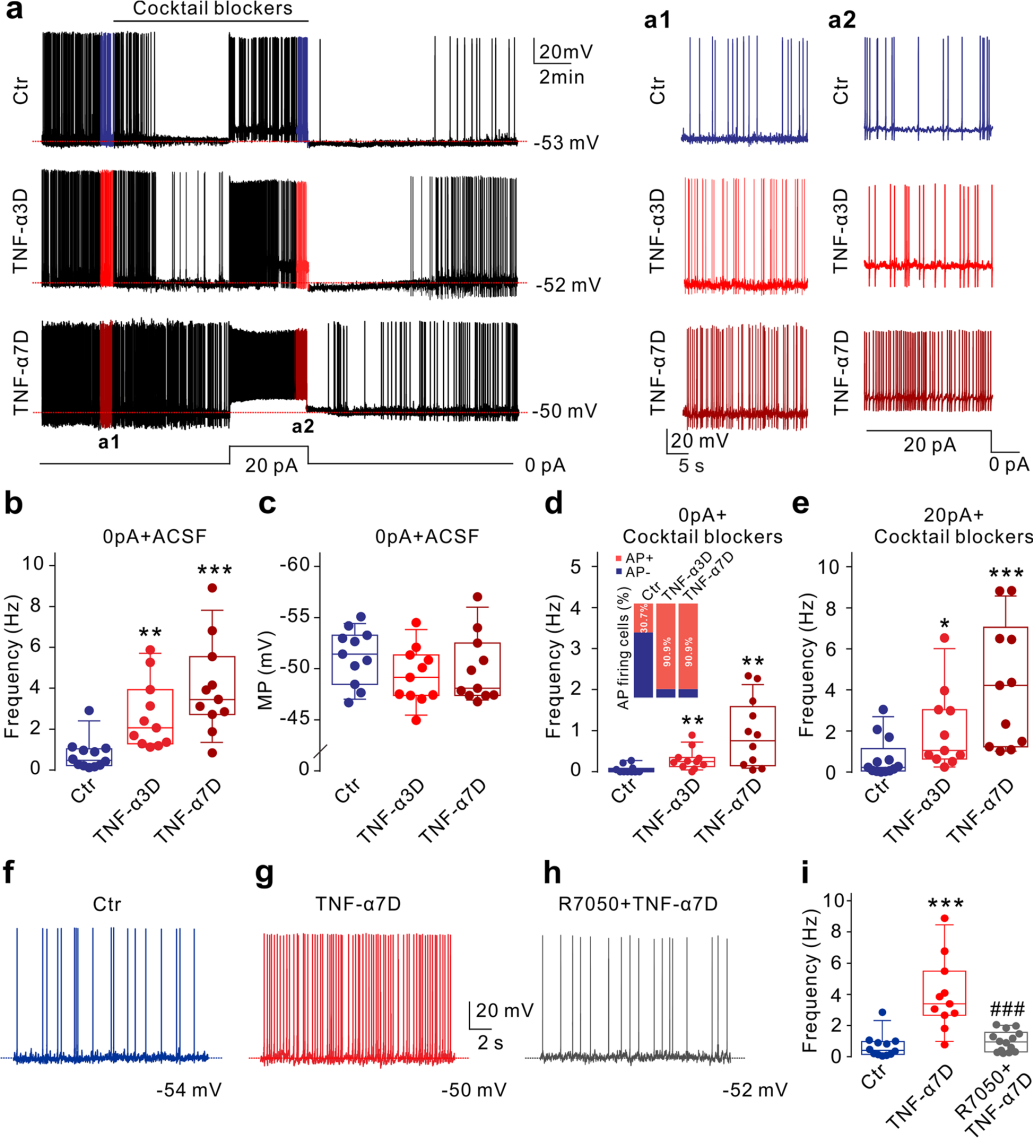
TNF-α induces hyperexcitability of RGCs through activating TNFR1. **a** Representative traces show spontaneous firing and evoked action potentials (AP) that were recorded in three different RGCs in rat retinal slices obtained from control (Ctr), 3 days (TNF-α3D) and 7 days (TNF-α7D) after intravitreal TNF-α injections. (a1) and (a2) are those from the recordings in **a** in a faster time scale. **b**, **c** Summary data show the changes in frequency (**b**) and membrane potential (MP) (**c**) of spontaneous firing in RGCs under different conditions. **d** Summary data show the changes in the frequency of spontaneous firing in RGCs under different conditions when synaptic transmissions were blocked. The insert shows the percentage of AP firing cells in different groups. **e** Summary data show the changes in the frequency of evoked AP under different conditions. *n* = 11 for each group. **f–h** Representative traces show spontaneous firing that was recorded in three different RGCs in rat retinal slices obtained from Ctr, TNF-α7D, and R7050+TNF-α7D groups, respectively. **i** Summary data show that the TNFR1 inhibitor R7050 eliminated TNF-α-induced increase of spontaneous firing frequency. *n* = 12, 11, 14, respectively. Data are represented as median and interquartile range. *P < 0.05, **P < 0.01, and ***P < 0.001 *vs.* Ctr; ^**###**^*P* < 0.001 *vs.* TNF-α7D

Since IOP elevation-induced hyperexcitability of RGCs in COH retinas could be reversed by TNF-α and NO blockers [28], we first examined whether TNF-α could induce RGC hyperexcitability in normal rats. Soluble TNF-α (5 ng/ml, 2 μl) was intravitreally injected, and retinal slices were made for electrophysiological recordings at 3 and 7 days (TNF-α3D and TNF-α7D) after the injections. In this study, all recordings in retinal slices were made on RGCs with a soma diameter of 10–15 μm, including both ON and OFF type cells, which were identified by their dendrite distribution in the IPL labeled by Alexa Fluor 488 and by negative current injections [38]. In previous studies [28, 38], we have shown that there were no significant differences between these two types of RGCs in both the frequency of spontaneous firing and the membrane potential under our recording conditions. Additionally, it was reported that at an early stage (2 weeks) of IOP elevation, dendritic pruning, and a transient increase in axon firing of RGCs in response to the preferred light stimulus were observed, which was independent of ON and OFF RGCs [6]. Therefore, data obtained from both types of RGCs were pooled in this study. As shown in Fig. 1a, RGCs displayed spontaneous firing under the current-clamped condition in normal rats (control, Ctr). Perfusion of the cocktail synaptic blockers, including bicuculline (10 μM), strychnine (10 μM), CNQX (10 μM), and D-APV (50 μM), almost completely blocked the spontaneous firing of the cell, similar to our previous report [38]. In the presence of cocktail synaptic blockers, current injection (+20 pA) could evoke the cell to fire action potentials (APs). In TNF-α injected retinas, the frequencies of spontaneous firing were significantly increased to 2.00 Hz (1.22, 3.87) (*n* = 11, *P* = 0.0018) and 3.40 Hz (2.66, 5.49) (*n* = 11, *P* < 0.001) in TNF-α3D and TNF-α7D groups, respectively, from the control value of 0.41 Hz (0.15, 0.97) (*n* = 12) (Fig. 1a, b). Although the membrane potentials (MP) of RGCs showed a trend toward depolarization in TNF-α injected retinas, there was no significant difference between control and TNF-α injected groups (Fig. 1a, c). When the synaptic transmissions were blocked, more than 90% of RGCs still showed spontaneous firing in both TNF-α3D and TNF-α7D groups, and the percentage of AP firing cells was higher than that of control (30.7%) (Fig. 1d, inset). Additionally, the frequencies of spontaneous firing were significantly increased (TNF-α3D: 0.25 Hz (0.13, 0.35), *n* = 11, *P* = 0.0054; TNF-α7D: 0.76 Hz (0.15, 1.59), *n* = 11, *P* = 0.0026), as compared to the control group (0.00 Hz (0.00, 0.08), *n* = 11) (Fig. 1d). Similarly, the frequencies of evoked AP were increased to 1.06 Hz (0.63, 3.04) (*n* = 11, *P* = 0.0353) and 4.22 Hz (1.24, 6.50) (*n* = 11, *P* = 0.0007) in TNF-α3D and TNF-α7D groups, respectively, from the control value of 0.23 Hz (0.05, 1.15) (*n* = 13) (Fig. 1e). Since soluble TNF-α-mediated effects are mainly through binding to TNFR1 [18–20], we examined whether TNF-α-induced RGC hyperexcitability is mediated by this receptor. Intravitreal co-injection of R7050 (10 μM, 2 μl), an inhibitor of TNFR1 signaling [39], blocked the TNF-α-induced increase in frequencies of spontaneous firing (Ctr: 0.41 Hz (0.15, 0.97), *n* = 12; TNF-α7D: 3.40 Hz (2.66, 5.49), *n* = 11, *P* < 0.001 *vs.* Ctr; R 7050+TNF-α7D: 0.95 Hz (0.30, 1.56), *n* = 14, *P* < 0.001 *vs.* TNF-α7D alone) (Fig. 1f–i). These results suggest that TNF-α may directly act on RGCs to induce cell hyperexcitability through activating TNFR1.

**Fig. 2:**
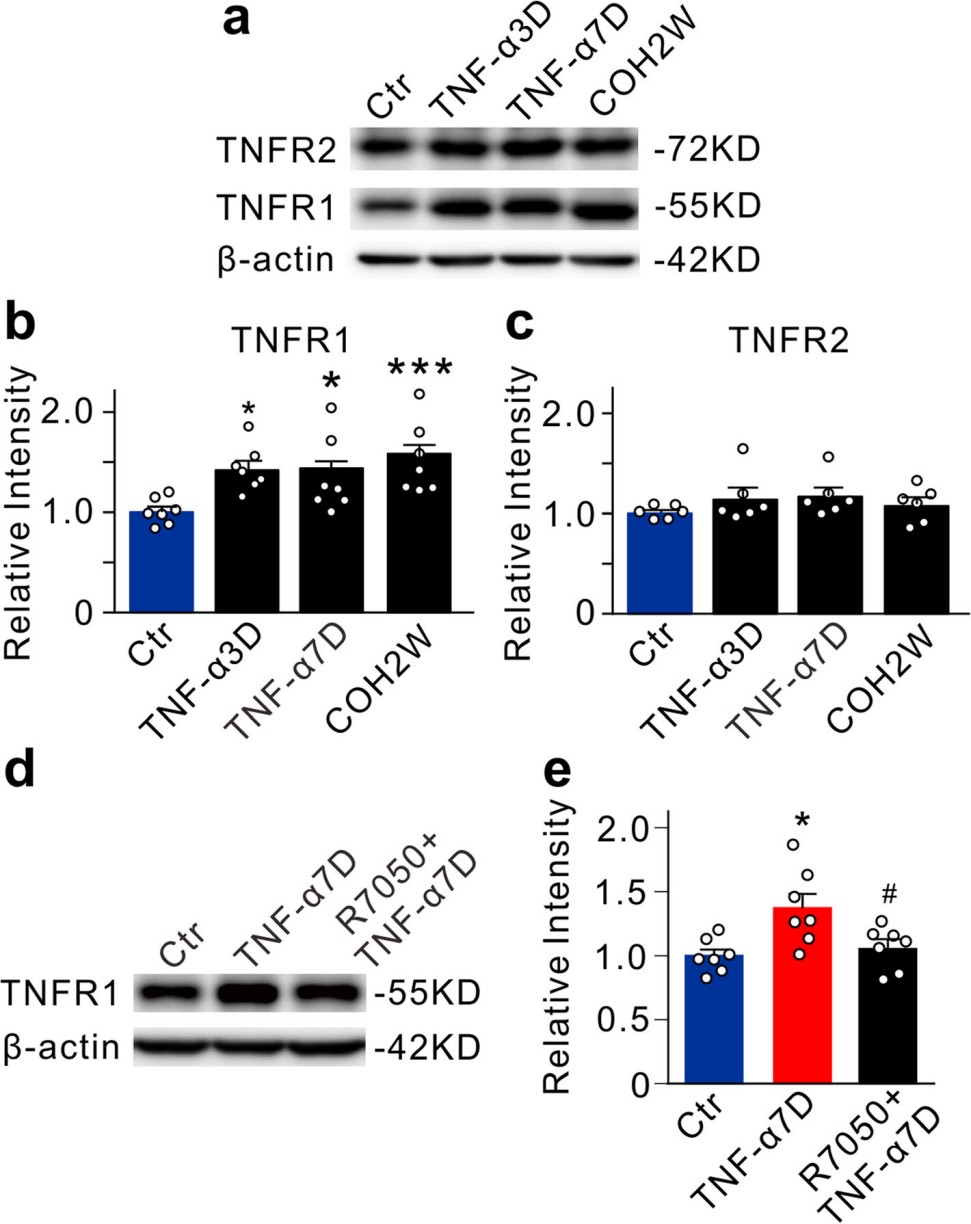
TNF-α induces upregulation of TNFR1 expression. **a** Representative immunoblots show the changes of TNFR1 and TNFR2 protein levels in control (Ctr), TNF-α3D, TNF-α7D, and COH2W retinas. **b**, **c** Bar charts summarize the average densitometric quantification of TNFR1 (**b**) and TNFR2 (**c**) in different groups. *n* = 6 for each group. **d**, **e** Representative immunoblots and the relative protein levels of TNFR1 in control (Ctr), TNF-α7D, and R 7050+TNF-α7D retinas. *n* = 7 for each group. *P < 0.05, and ***P < 0.001 *vs.* Ctr; ^**#**^*P* < 0.05 *vs.* TNF-α7D

We also determined whether intravitreal injection of TNF-α may change the expression of TNF-α receptors. As shown in Fig. 2, in TNF-α-injected retinas, the protein levels of TNFR1 expression were significantly increased to 141.9 ± 8.2% of control (*n* = 7, *P* = 0.0147) and 143.9 ± 12.4% of control (*n* = 7, *P* = 0.0104) in TNF-α3D and TNF-α7D groups, respectively. As a positive control, the TNF-α protein levels were increased to 158.5 ± 11.7% of control in COH retinas at week 2 (COH2W) (*n* = 7, *P* < 0.001) (Fig. 2a, b). In contrast, the protein levels of TNFR2 expression kept unchanged in both TNF-α-injected and COH retinas (Fig. 2a, c). Furthermore, co-injection of R7050 abolished the TNF-α-induced upregulation of TNFR1 expression (TNF-α7D: 137.1 ± 11.1% of control, *n* = 7, *P* = 0.0147 *vs.* Ctr; R 7050+TNF-α7D: *n* = 7, 105.3 ± 7.8% of control, *P* = 0.0377 *vs.* TNF-α7D) (Fig. 2d, e).

### TNF-α selectively upregulates Nav1.6 currents in RGCs

**Fig. 3:**
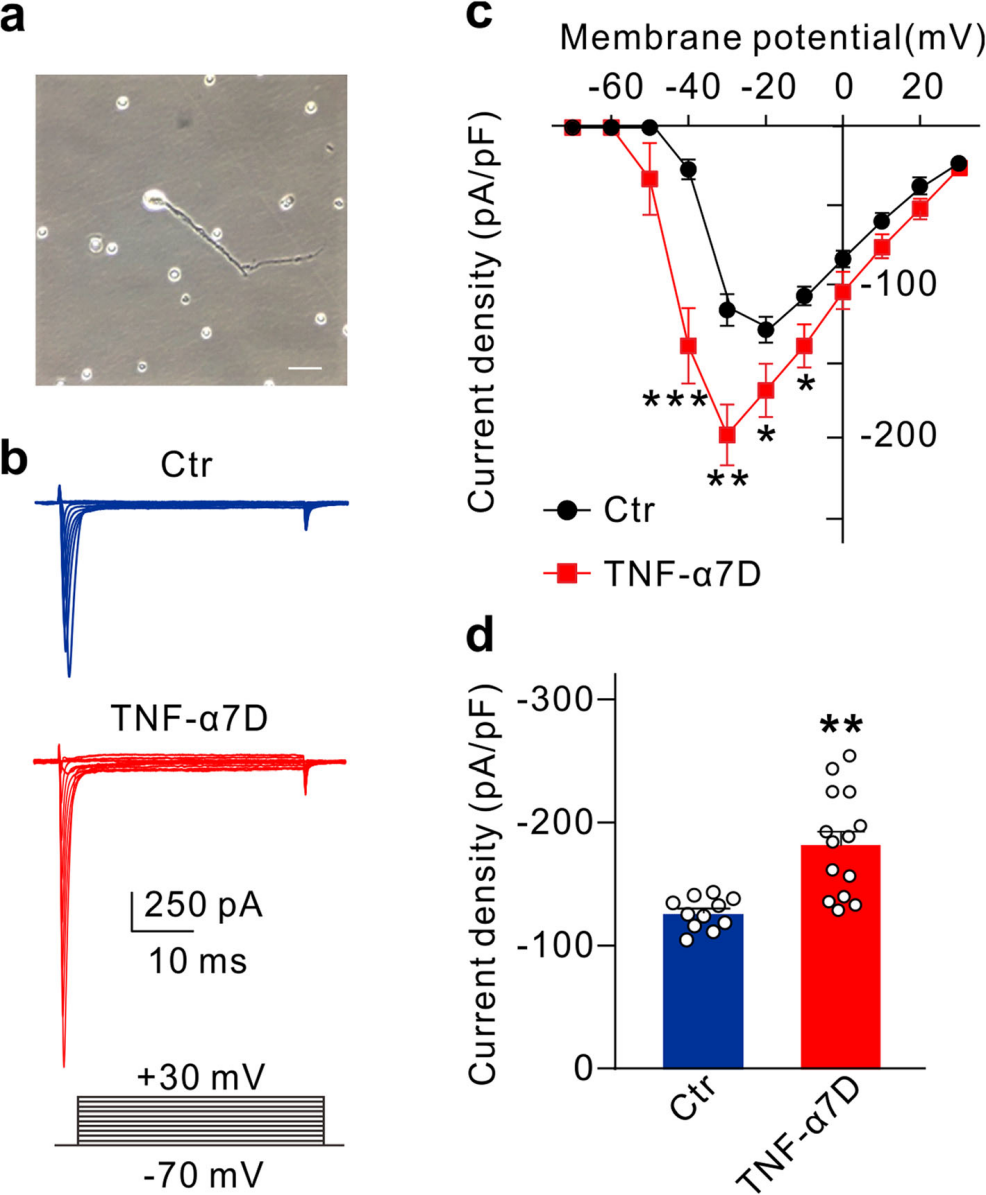
TNF-α voltage-dependently increases Na^+^ current density in RGCs. **a** Micrograph showing a typical acutely isolated RGC for recording. **b** Representative traces show Na^+^ currents recorded in acutely isolated RGCs from control (Ctr) and TNF-α7D retinas. The holding potentials of RGCs were set at −70 mV and the currents were evoked in the range of −70 to +30 mV with steps of 10 mV. **c** Current-voltage relationship curves of Na^+^ currents in RGCs of Ctr and TNF-α7D retinas. *n* = 11 and 14 for Ctr and TNF-α7D groups, respectively. **d** Summary data show the peak current densities of Na^+^ channels at −30 mV in RGCs of Ctr and TNF-α7D retinas. *n* = 11 and 14 for Ctr and TNF-α7D groups, respectively. *P < 0.05, **P < 0.01, and ***P < 0.001 *vs.* Ctr

Previous studies have shown that TNF-α could regulate VGSCs in neurons, thus influencing neuronal excitability [40–42]. We tested the possibility that VGSCs are involved in TNF-α-induced RGC hyperexcitability. Whole cell Na^+^ currents were recorded in acutely isolated RGCs (Fig. 3a) from control and TNF-α intravitreally injected (TNF-α7D) rats. As compared with the controls, Na^+^ current densities in the TNF-α7D group were significantly and voltage-dependently increased (Fig. 3b, c). For example, at −30 mV peak Na^+^ current density was increased to −180.8 ± 14.7 pA/pF (*n* = 14; *P* = 0.0032) from the control value of −124.6 ± 4.45 pA/pF (*n* = 11) (Fig. 3d).

**Fig. 4:**
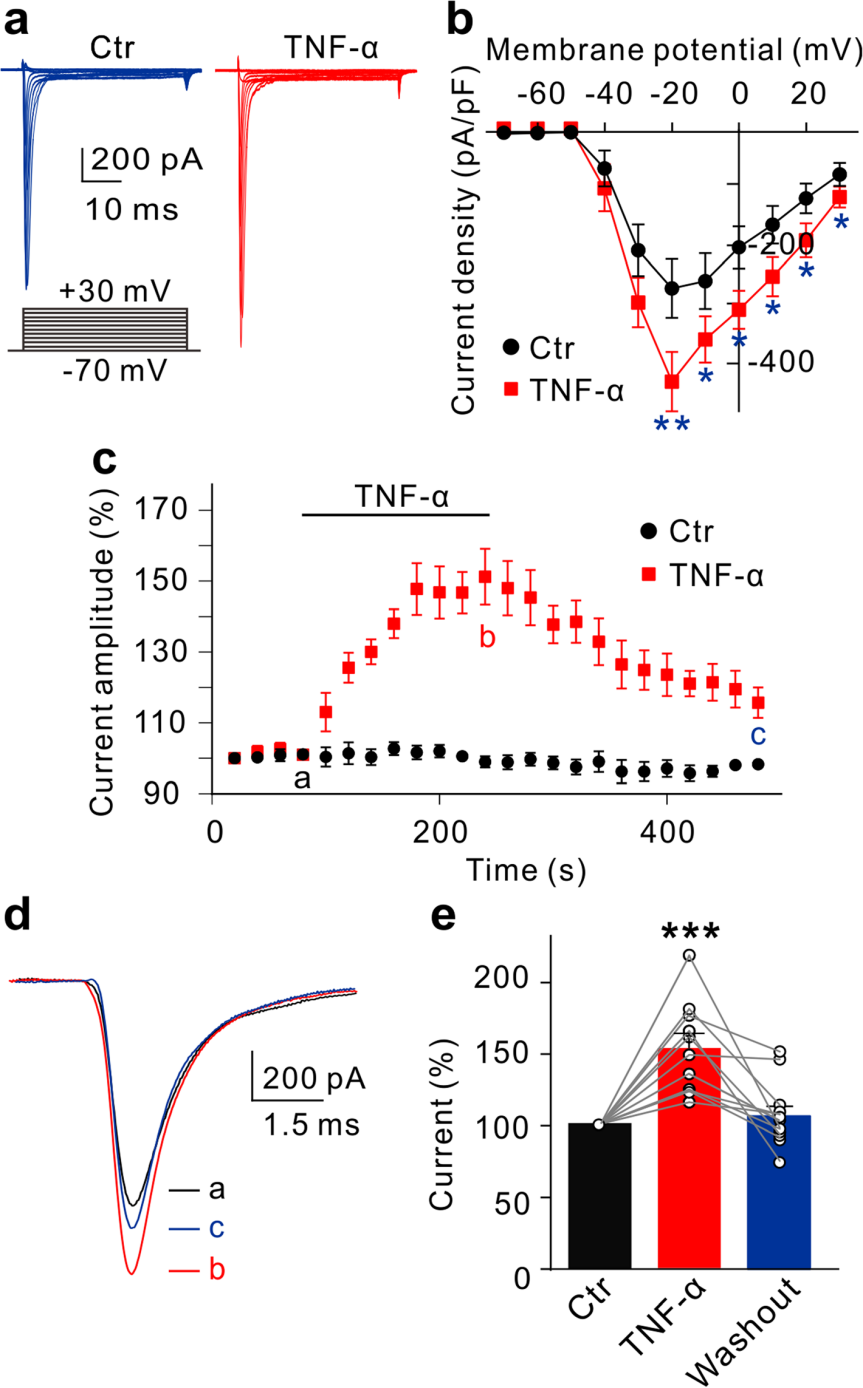
TNF-α increases Na^+^ currents in RGCs of normal retinas. **a** Representative traces show Na^+^ currents recorded in an RGC before (control, Ctr) and after TNF-α (5 ng/ml) application. **b** Current-voltage relationship curves of Na^+^ currents in RGCs before (Ctr) and after TNF-α application. *n* = 11. **c** Time courses of Na^+^ currents at −20 mV in Ctr (*n* = 7) and TNF-α application groups (*n* = 8). **d** Representative Na^+^ current traces recorded in an RGC at −20 mV in the TNF-α group at different time points as shown in figure c panel. **e** Summary data show the changes in relative peak Na^+^ currents (at −20 mV) under different conditions. *n* = 11. *P < 0.05, **P < 0.01, and ***P < 0.001 *vs.* Ctr

**Fig. 5:**
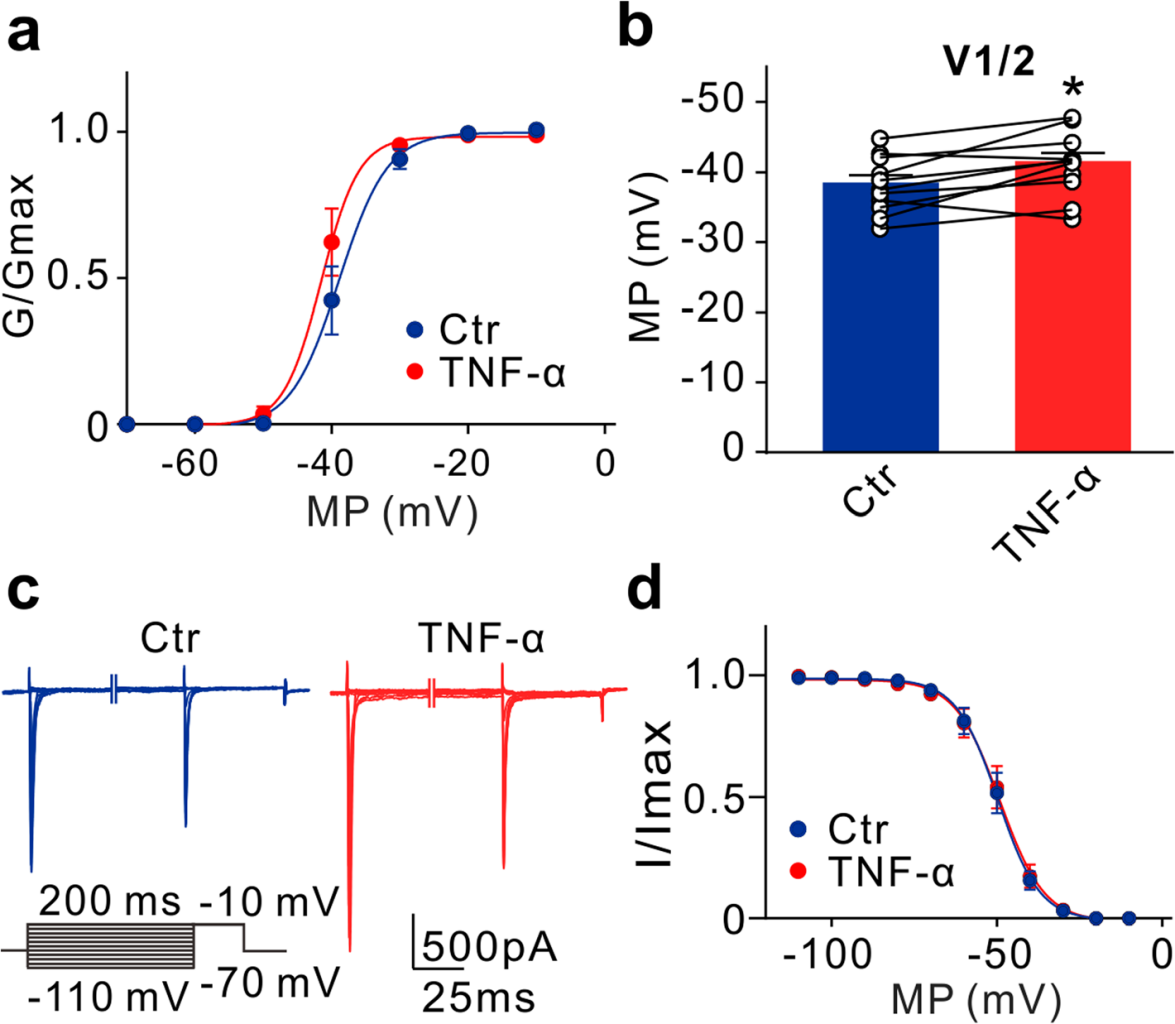
TNF-α increases the activation probability of Na^+^ currents. **a** Activation curves of Na^+^ currents in RGCs before and after TNF-α application. **b** Bar chart summarizes the changes in the *V*_*1/2*_ of Na^+^ currents before and after TNF-α application. *n* = 12; *P < 0.05 *vs.* Ctr. **c** Representative Na^+^ currents recorded in an RGC before and after TNF-α application. The cell was given a 200 ms pre-pulse from a holding potential of −70 mV to different membrane potentials and then depolarized to −10 mV. **d** Inactivation curves of Na^+^ currents before and after TNF-α application. *n* = 6. Both the activation and inactivation curves were fitted with the Boltzmann equation

Upregulation of Na^+^ currents induced by intravitreal injection of TNF-α may be mediated by directly regulating ion channels or/and increasing ion channel protein expression. We first tested the effect of TNF-α on VGSCs in acutely isolated RGCs from normal rats. Figure 4a shows the representative Na^+^ currents recorded in an RGC before and after TNF-α (5 ng/ml) application. TNF-α significantly and voltage-dependently enhanced peak Na^+^ current densities (Fig. 4b). In addition, the effect of TNF-α on Na^+^ currents was reversible. As shown in Fig. 4c, at −20 mV, stable current recordings could be kept for almost 8 min, perfusion of TNF-α reversibly increased the current amplitudes to 152.2 ± 10.5% of control (*n* = 11, *P* < 0.001), and washout pushed the currents almost to control levels (Fig. 4d, e). We also examined the changes of activation and inactivation curves of Na^+^ currents in RGCs before and after TNF-α application. As shown in Fig. 5a, the activation curve of Na ^+^ currents was shifted toward hyperpolarization direction after TNF-α application, with a *V*_1/2_ value being −41.20 ± 1.4 mV (*n* = 10, *P* = 0.0102) that was significantly different from control value (−38.12 ± 1.21 mV, *n* = 10) (Fig. 5b). However, TNF-α did not change the inactivation curve (control: *V*_1/2_ = −49.5 ± 2.1 mV, *n* = 7; TNF-α: *V*_1/2_ = −49.6 ± 2.3 mV, *n* = 7, *P* = 0.7648) (Fig. 5c, d). These results suggest that TNF-α could directly enhance Na^+^ currents and increase the activation probability of the channels.

**Fig. 6:**
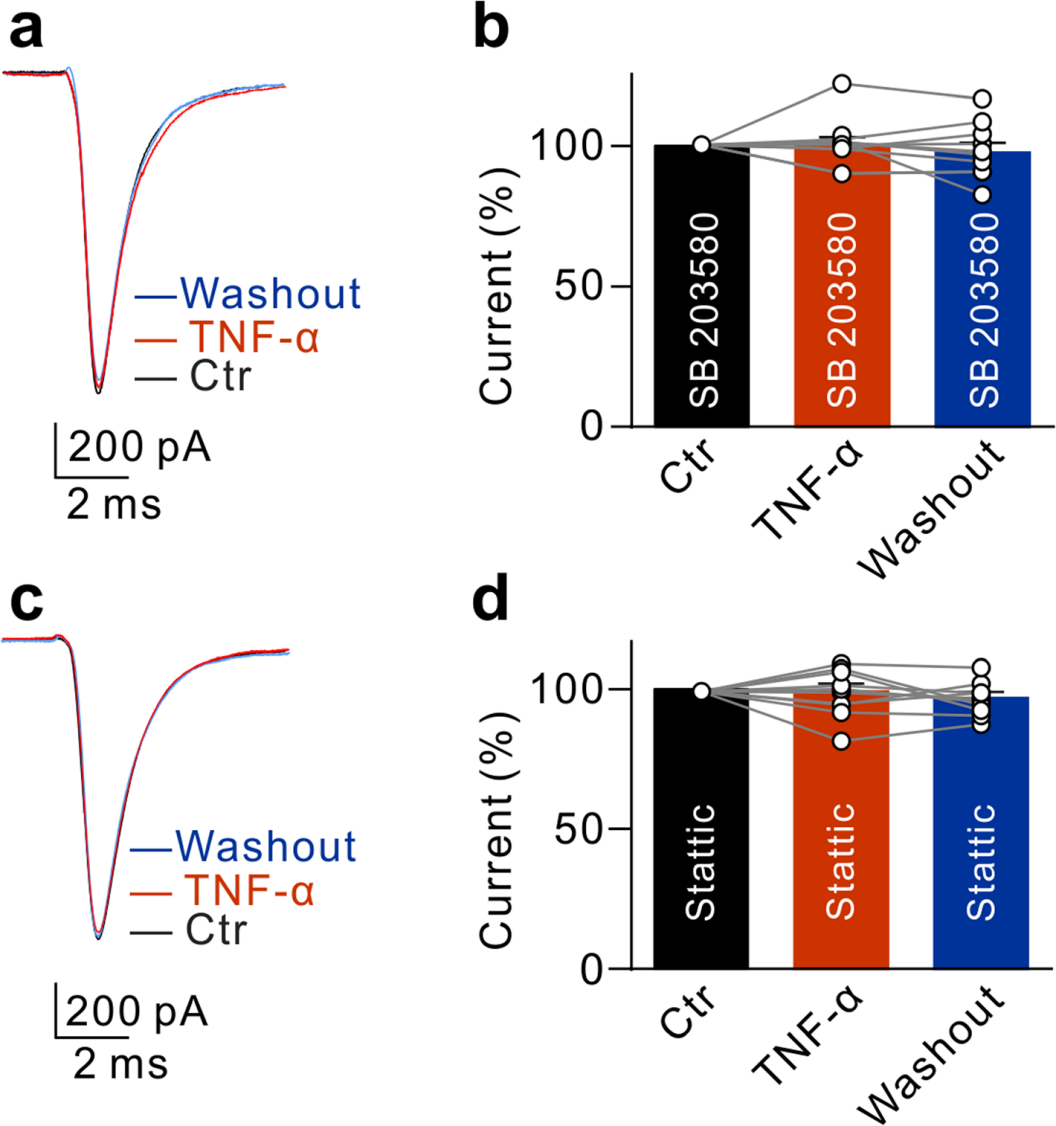
p38 MAPK and STAT3 pathways mediate TNF-α-induced upregulation of Na^+^ currents in RGCs. **a**, **b** Sample traces recorded in an RGC at −20 mV show that pre-incubation of the p38 MAPK inhibitor SB203580 blocked the TNF-α-induced upregulation of peak Na^+^ currents (**a**), and summary data are shown in **b**. *n* = 10. **c**, **d** Sample traces recorded another RGC at −20 mV show that pre-incubation of the STAT3 inhibitor stattic blocked the TNF-α-induced upregulation of peak Na^+^ currents (**c**), and summary data are shown in **d**. *n* = 10

It was reported that p38 MAP kinase (p38 MAPK) and signal transducer and activator of transcription 3 (STAT3) signaling pathways were involved in TNF-α-mediated effects [43, 44]. The mechanisms underlying the TNF-α-induced enhancement of Na^+^ currents were explored. Figure 6a, b shows that TNF-α-induced upregulation of Na^+^ currents in RGCs was eliminated when the cells were pre-incubated with SB203580 (10 μM), a p38 MAPK inhibitor, for 30 min (100.4 ± 2.8% of control, *n* = 10, *P* = 0.8777). Similar results were obtained when RGCs were pre-incubated with Stattic (10 μM), a STAT3 inhibitor (99.4 ± 2.6% of control, *n* = 10, *P* = 0.8148) (Fig. 6c, d). These results indicate that TNF-α upregulates Na^+^ currents in RGCs through p38 MAPK and STAT3 signaling pathways.

**Fig. 7:**
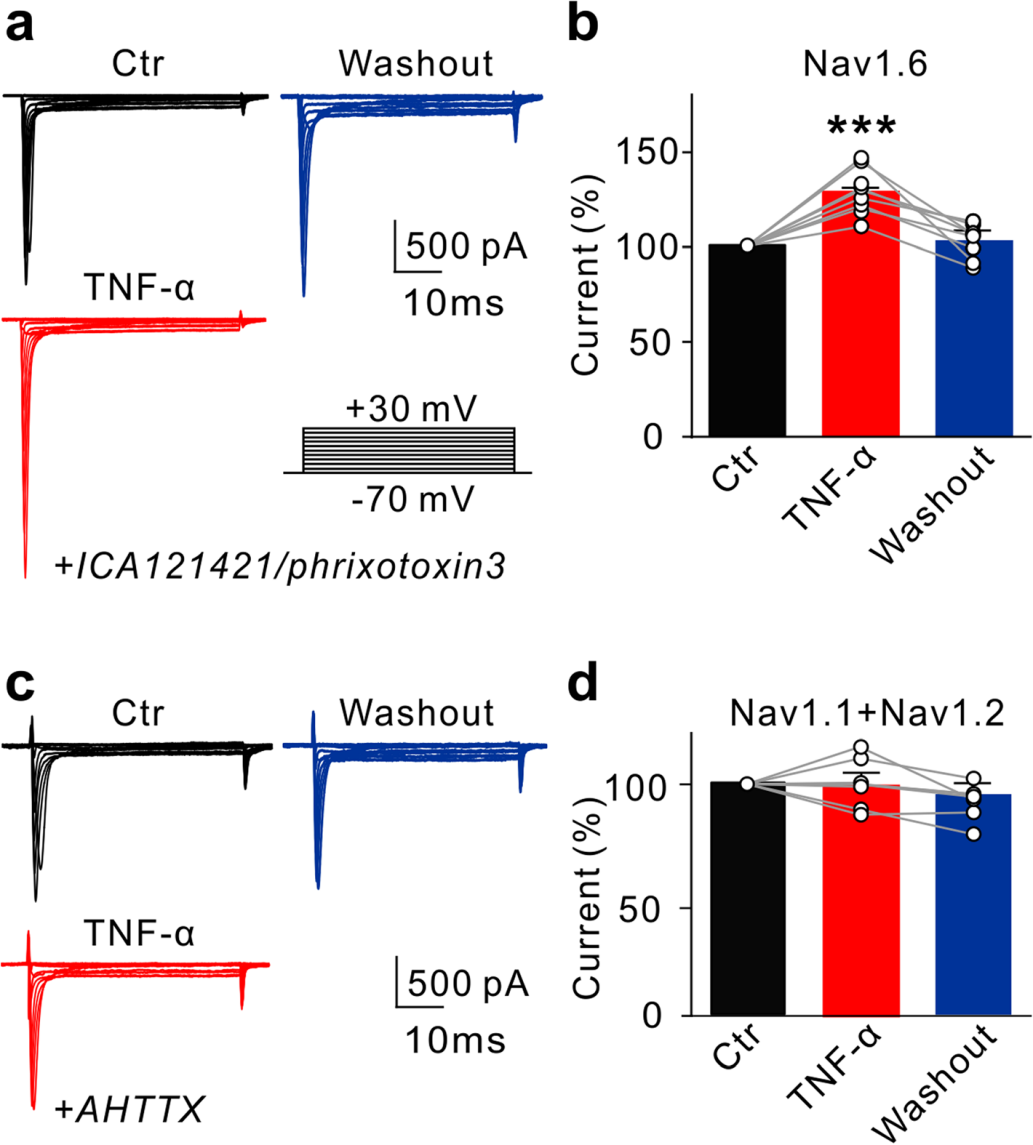
TNF-α selectively upregulates Nav1.6 currents in rat RGCs. **a** Representative current traces recorded from an RGC show the changes of peak Nav1.6 currents before and after TNF-α application in the presence of the Nav1.1 blocker ICA12142 (1 μM) and the Nav1.2 blocker Phrixotoxin3 (10 nM). **b** Bar charts summarize the changes of Nav1.6 current amplitudes at −20 mV before and after TNF-α perfusion. Note that TNF-α enhanced Nav1.6 peak currents. *n* = 9. ***P < 0.001 *vs.* Ctr. **c** Representative traces show the changes in Nav1.1 and Nav1.2 mixed currents recorded in an RGC before and after TNF-α application in the presence of the Nav1.6 blocker AHTTX (100 nM). **d** Bar charts summarize the changes of mixed Nav1.1 and Nav1.2 current amplitudes before and after TNF-α application. *n* = 7

RGCs express functional Nav1.1, Nav1.2, and Nav1.6 Na^+^ channels, and Nav1.6 is the predominant one [45–47]. We examined which subtype(s) may be modulated by TNF-α. As shown in Fig. 7a, an RGC was pre-incubated with the Nav1.1 blocker ICA121421 (1 μM) and the Nav1.2 blocker phrixotoxin3 (10 nM). In the presence of these blockers, perfusion of TNF-α still increased Na^+^ currents. The average current amplitudes were increased to 128.3 ± 4.3% of control (*n* = 8, *P* < 0.001) (Fig. 7b). In contrast, when RGCs were pre-incubated with the Nav1.6 blocker 4,9-anhydrotetrodotoxin (AHTTX, 100 nM), perfusion of TNF-α no longer changed the currents (100.5 ± 4.7% of control, *n* = 6, *P* = 0.9168) (Fig. 7c, d). These results suggest that TNF-α may selectively enhance Nav1.6 currents.

**Fig. 8:**
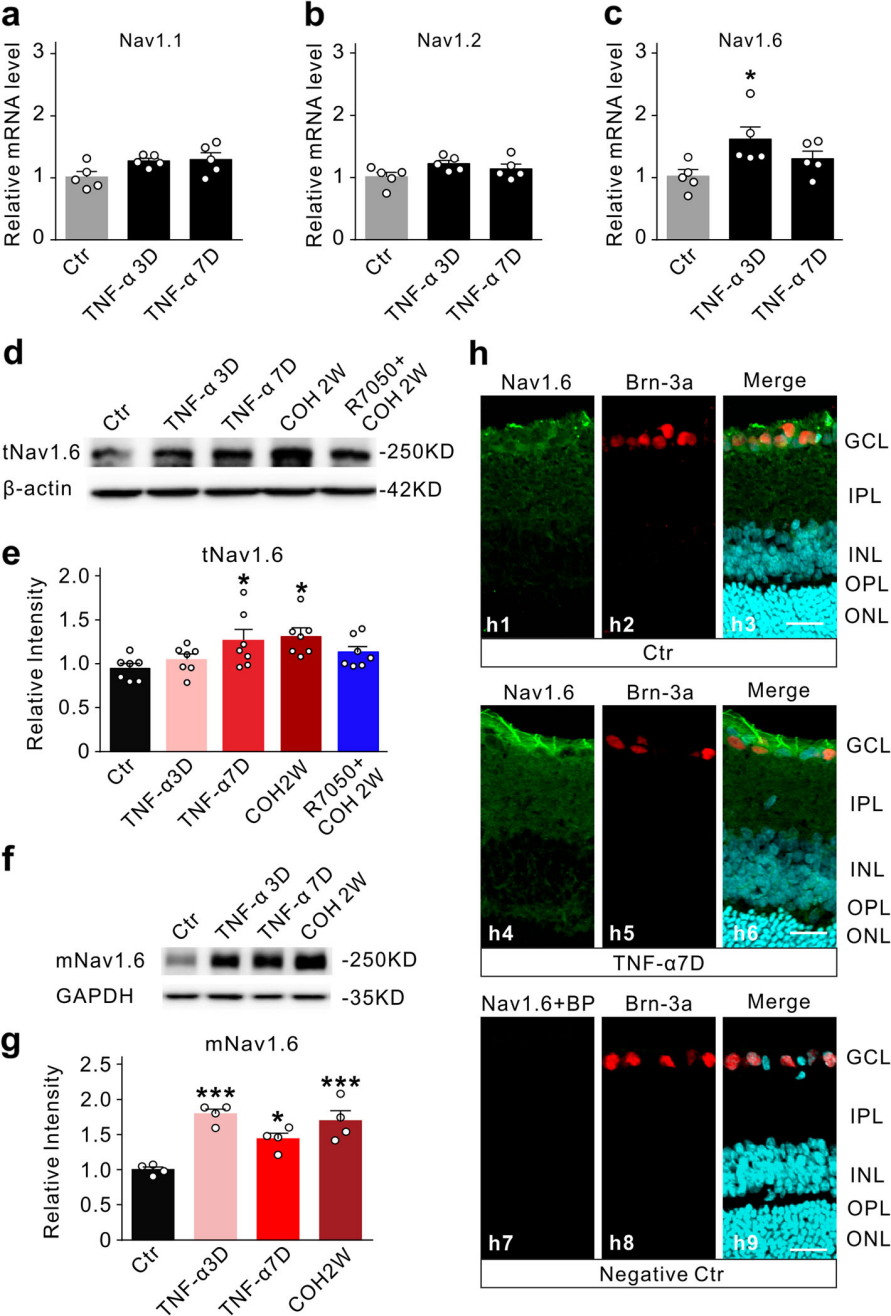
TNF-α increases the expression of Nav1.6 in the retina. **a–c** Bar charts show the relative mRNA levels of Nav1.1, Nav1.2, and Nav1.6 in Ctr (**a**), TNF-α3D (**b**), and TNF-α7D (**c**) groups. *n* = 5 for each group. **d** Representative immunoblots show the changes in total Nav1.6 (tNav1.6) expression in control (Ctr), TNF-α3D, TNF-α7D, and COH2W without or with R7050 retinal extracts. **e** Bar charts summarize the average densitometric quantification of immunoreactive bands of tNav1.6 under different conditions as shown in **d**. *n* = 6 for each group. All data are normalized to their corresponding β-actin and then to Ctr. **f** Representative immunoblots show the changes in Nav1.6 expression in membrane components (mNav1.6) in control (Ctr), TNF-α3D, TNF-α7D, and COH2W retinal extracts. *n* = 4 for each group. All data are normalized to their corresponding GAPDH and then to Ctr. *P < 0.05 and ***P < 0.001 *vs.* Ctr. **h** Immunofluorescence labeling showing Nav1.6 expression in rat retinal vertical slices taken from the normal saline-injected retina (Ctr, h1-h3) and TNF-α-injected retinas on 7 days after the injection (TNF-α7D, h4-h6). h7-h9: double immunofluorescence staining showing Nav1.6 expression when the Nav1.6 antibody was pre-absorbed with its blocking peptide (BP). Note that the enhanced expression of Nav1.6 in the axon-like structures in the TNF-α7D group. Scale bars = 20 μm. GCL, ganglion cell layer; IPL, inner plexiform layer; INL, inner nuclear layer; OPL, outer plexiform layer; ONL, outer nuclear layer

We then examined whether TNF-α injection may change the protein expression levels of Na^+^ channels. The qPCR analysis revealed that the mRNA levels of Nav1.6 were significantly increased at 3 days after the TNF-α injection, then declined at 7 days, while the mRNA levels of Nav1.1 and Nav1.2 were kept unchanged (Fig. 8a–c). Therefore, we examined Nav1.6 protein expression in TNF-α injected retinas. The total protein level of Nav1.6 (tNav1.6) in the TNF-α3D group was not changed as compared with the control (104.5 ± 5.7% of control, *n* = 7, *P* = 0.7949); however, it was increased to 126.5 ± 12.39% of control (*n* = 7, *P* = 0.0298) in the TNF-α7D group. As a positive control, the total protein level of Nav1.6 in COH2W was increased to 130.9 ± 8.9% of control (*n* = 7, *P* = 0.0118), which was reversed by pre-injecting the TNFR1 blocker R7050 (10 μM) (113.4 ± 6.1% of control, *n* = 7, *P* = 0.3053 *vs.* control) (Fig. 8d, e). Since Na^+^ channels expressed in the cell membrane are functional, the protein levels of Nav1.6 in the membrane component (mNav1.6) were further examined. Figure 8f, g clearly showed that intravitreal injection of TNF-α significantly upregulated mNav1.6 expression (TNF-α3D: 179.3 ± 6.9% of control, *n* = 4, *P* < 0.001; TNF-α7D: 143.9 ± 8.2% of control, *n* = 4, *P* = 0.0253). In COH retinas, mNav1.6 expression was also increased to 169.6 ± 14.6% of control, *n* = 4, *P* < 0.001). Consistently, double immunostaining showed that Nav1.6 was primarily expressed in the ganglion cell layer (GCL) and the inner plexiform layer (IPL), and intravitreal injection of TNF-α increased Nav1.6 expression, especially in the RGC axon-like structures (Fig. 8h).

**Fig. 9:**
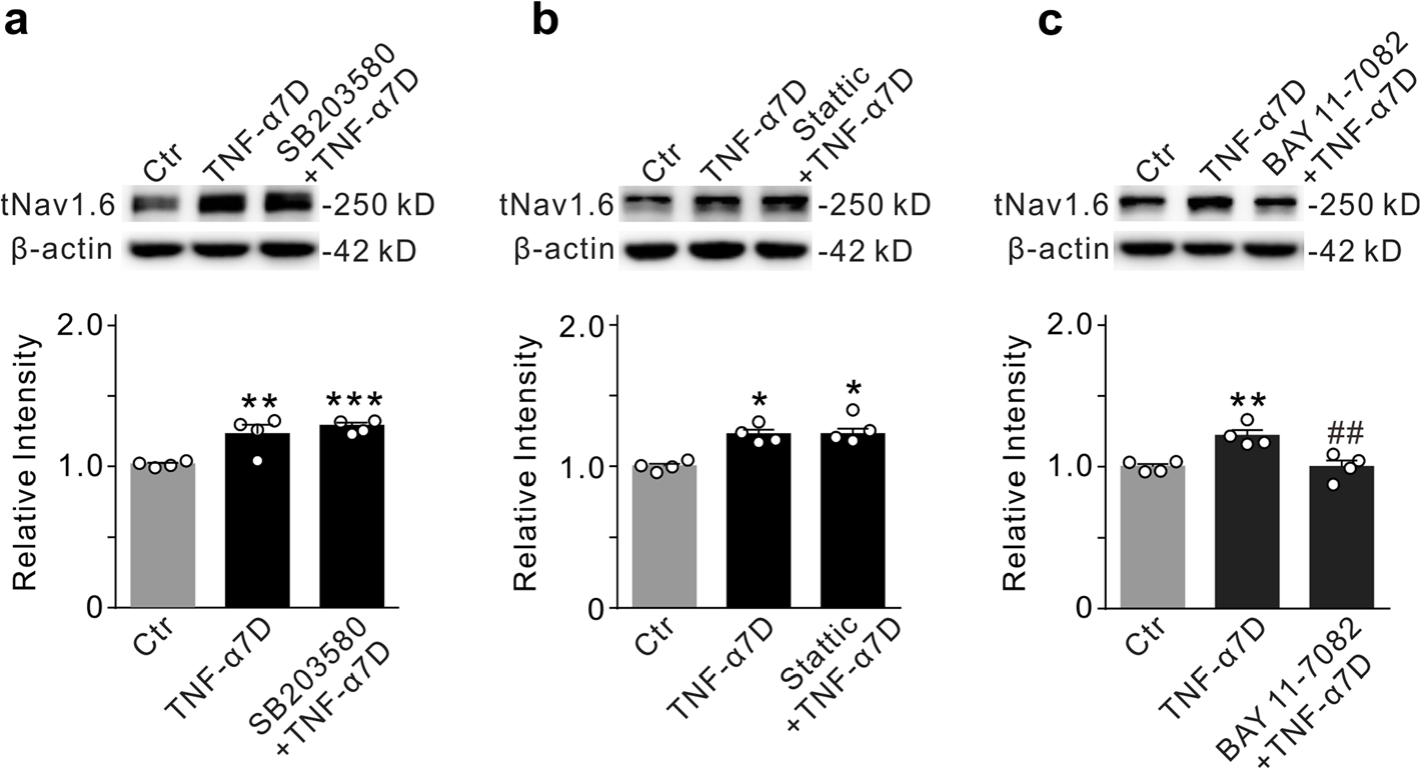
NF-κB signaling pathway mediates TNF-α-induced upregulation of Nav1.6 expression. **a** Representative immunoblots show the changes in tNav1.6 expression in control (Ctr), TNF-α7D, and SB203580+TNF-α7D retinal extracts (upper panel), and summary data are shown in the lower panel. **b** Representative immunoblots (upper panel) and summary data (lower panel) of tNav1.6 protein levels in Ctr, TNF-α7D, and stattic+TNF-α7D groups. **c** Representative immunoblots (upper panel) and summary data (lower panel) of tNav1.6 protein levels in Ctr, TNF-α7D, and BAY 11-7082+TNF-α7D groups. All data are normalized to their corresponding β-actin and then to Ctr. *n* = 4 for each group. *P < 0.05, **P < 0.01, and ***P < 0.001 *vs.* Ctr; ^**##**^*P* < 0.01 *vs.* TNF-α7D

Signaling pathways involved in TNF-α-induced increase in Nav1.6 expression were also examined. Unexpectedly, p38 MAPK and STAT3 signaling pathways were not involved in the upregulation of Nav1.6 expression since intravitreal injections of the p38 MAPK inhibitor SB203580 or the STAT3 inhibitor stattic failed to block the TNF-α-induced effects on Nav1.6 expression (SB203580+TNF-α7D: *n* =4, *P* = 0.6528 *vs.* TNF-α7D alone; stattic+TNF-α7D: *n* =4, *P* = 0.8004 *vs.* TNF-α7D alone) (Fig. 9a, b). In contrast, intravitreal injection of the NF-κB inhibitor BAY 11-7082 (10 μM) could efficiently block the upregulated expression of Nav1.6 induced by TNF-α (99.9 ± 4.6% of control, *n* =4, *P* = 0.0053 *vs*. 121.9 ± 4.0% of control in TNF-α7D alone, *n* = 4) (Fig. 9c).

### TNF-α induces RGC apoptosis

**Fig. 10:**
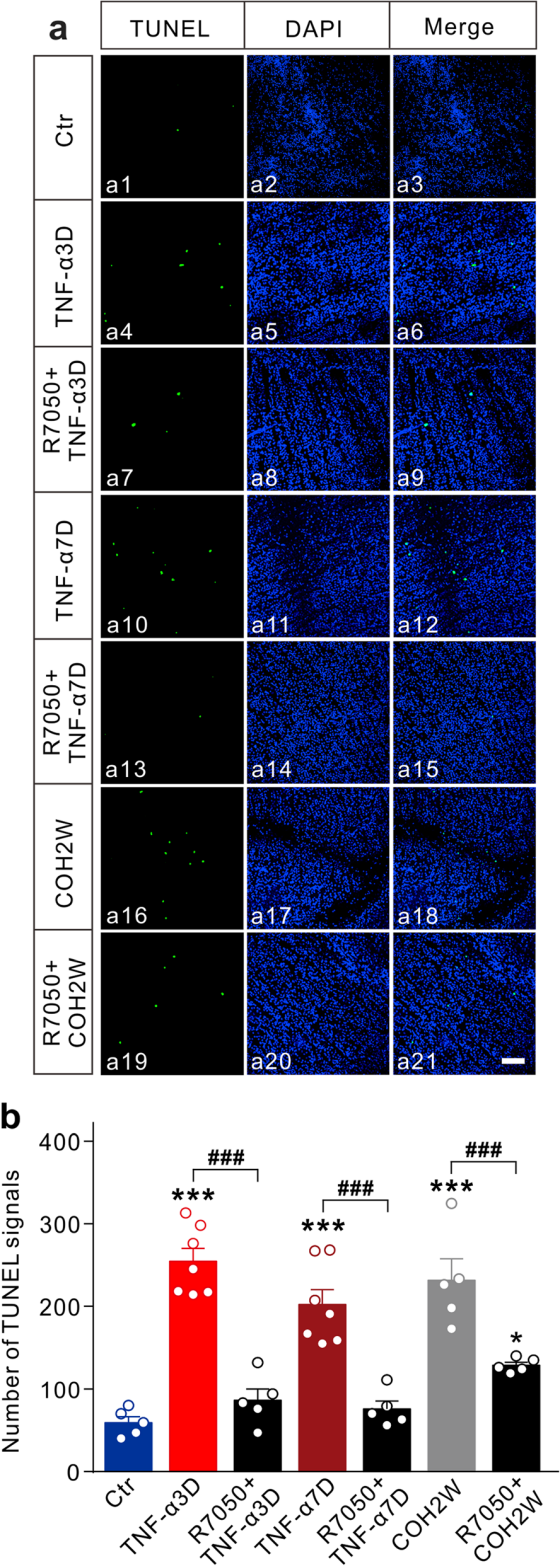
Inhibition of TNFR1 reduces RGC apoptosis in TNF-α injected and COH retinas. **a** Representative images of TUNEL staining detection of apoptotic RGCs in Ctr (a1), TNF-α3D (a4), R7050+TNF-α3D (a7), TNF-α7D (a10), R7050+TNF-α7D (a13), COH2W (a16), and R7050+COH2W (a19) groups. R7050 (10 μM, 2 μl) was intravitreally injected 1 day before TNF-α injection or COH operation. All images were taken from the whole-flat mounted retinas in the regions at angle 0°. a2, a5, a8, a11, a14, a17, and a20 were DAPI images. Merged images of TUNEL and DAPI were showed in a3, a6, a9, a12, a15, a18, and a21. Scale bar = 50 μm for all images. **b** Bar charts summarize the average number of TUNEL-positive signals in whole flat-mounted retinas in different groups. *n* = 5–7. *P < 0.05, **P < 0.01, and ***P < 0.001 *vs.* Ctr; ^**###**^*P* < 0.001

Finally, we explored the TNF-α-induced effects on RGC survival by using TUNEL-staining method. Representative TUNEL staining images captured from whole flat-mounted retinas under different conditions are shown in Fig. 10a. Intravitreal injection of TNF-α remarkably increased the number of TUNEL-positive RGCs to 254.4 ± 15.6 (*n* = 7, *P* < 0.001) and 202.0 ± 18.4 (*n*= 7, *P* < 0.001) in TNF-α3D and TNF-α7D groups, respectively, which could be reversed by co-injection of the TNFR1 blocker R7050 (R7050+TNF-α3D: 86.4 ± 13.8, *n* = 5, *P* < 0.001 *vs.* TNF-α3D group; R7050+TNF-α7D: 75.8 ± 9.6, *n* = 5, *P* < 0.001 *vs.* TNF-α7D group) (Fig. 10b). Similarly, pre-injection of R7050 (10 μM) could partially reduce the number of TUNEL-positive RGCs in COH retinas (128.8 ± 3.8, *n* = 5, *P* = 0.0295 *vs.* control; *P* = 0.0024 *vs.* COH2W group) (Fig. 10b).

## Discussion

### TNF-α-induced neuronal hyperexcitability contributes to RGC injury

It is commonly known that neuroinflammation plays a key role in the pathogenesis of glaucoma although the detailed mechanisms have not been completely elucidated [13, 48]. As one of the major inflammatory factors, TNF-α is released from the activated glial cells in glaucomatous retinas [49–51]. In this study, we demonstrated that TNF-α could induce RGC hyperexcitability, as evidenced by increased spontaneous firing and evoked AP frequencies of the cells, thus contributing to RGC injury in glaucoma. Changes in spontaneous firing may have resulted from the balance between excitatory and inhibitory inputs. When the synaptic transmissions were blocked, most of RGCs (> 90%) still showed spontaneous firing in the TNF-α-injected retinas, while a lesser number of RGCs fired AP spontaneously with a very low frequency in the control retinas, suggesting that TNF-α have a direct action on RGCs. Indeed, a number of studies have reported the enhanced excitability of RGCs in glaucomatous retinas [6, 28, 52, 53]. In an experimental glaucoma model, we have shown that IOP elevation resulted in RGC hyperexcitability, which may be mediated by inflammatory factors [28]. The present work provided further evidence that TNF-α was indeed involved. It should be noted that IOP elevation also induced a depolarized resting membrane potential in RGCs, in addition to hyperexcitability [28], while TNF-α had no significant effect on the membrane potential of RGCs. These results suggest that more factors may mediate RGC hyperexcitability in glaucoma, such as activated EphB/ephrinB signaling, group I metabotropic glutamate receptors, and G-protein-coupled dopamine receptors [28, 38, 54], which remains to be further investigated. It is noteworthy that there was evidence showing that IOP elevation in a mouse model induced a reduction in the frequency of spontaneous and light-evoked firing in RGCs when the IOP was elevated for 15 and 30 days [55]. For this apparent inconsistence, we speculated that it may be, at least in part, due to the recordings made at different stages of IOP elevation. Our recordings were made at an earlier stage (3–7 days after TNF-α injection). Progressive IOP elevation may result in RGC dendritic damage and loss of excitatory synapses at 15 and 30 days [55], thus reducing spontaneous firing. Actually, the spontaneous firing of rat RGCs was dependent upon presynaptic inputs [38]. Moreover, Risner et al. showed that a transient increase of light-induced firing frequency in RGCs was observed at 2 weeks after IOP elevation, and the frequency was decreased at 4 weeks [6].

In addition, Margolis and Detwiler reported that ON and OFF RGCs showed distinct spiking properties in the mouse retina: resting activity of ON RGCs was dependent on tonic excitatory inputs, whereas OFF RGCs exhibited properties of pacemaker neurons and continued to fire in the absence of synaptic inputs [56]. However, previous studies and our present results showed that in rat retina, the frequency of spontaneous firing and the membrane potential did not show significant differences between ON and OFF subtypes of RGCs [28, 38, 57]. To explain this inconsistency, we speculated that ON and OFF RGCs in mice might receive different synaptic inputs and have distinct intrinsic properties.

It should be noted that in dissociated RGCs, most dendrites and part of the axon of RGC may be destroyed during the isolated process. In this study, we usually chose the RGC with axon for recordings. Since dendrites were destroyed in these cells, some ion channels mainly expressed in dendrites, such as hyperpolarization-activated cation channel (*I*_h_) and inwardly rectifying potassium channel (Kir) [38], were lost, which may change the excitability of RGCs. However, in this study, all recordings made in the dissociated RGCs were performed to record VGSC currents. Considering the fact that Nav1.6 VGSCs are mainly expressed in the soma and the axon, especially axon initial segment (AIS), it may be no significant influence on the results.

In the central nervous system (CNS), pleiotropic effects of TNF-α are mainly mediated by two signaling pathways, soluble TNF-α/TNFR1, and transmembrane TNF-α/TNFR2, which may lead to opposing outcomes, deleterious, or beneficial effects for neurons [18–22, 58]. Although we cannot precisely separate the functional outcomes of these two TNF-α signaling, it is possible that under pathological conditions, the dominant pro-inflammatory receptor TNFR1 plays an important role in neuronal injury. In this study, we showed that TNF-α-induced RGC hyperexcitability was mediated through activating TNFR1. Firstly, the TNFR1 inhibitor R7050 completely abolished the TNF-α-induced increase in spontaneous firing of RGCs in retinal slices. Secondly, the protein levels of TNFR1 in TNF-α injected and COH retinas were significantly upregulated, while TNFR2 expression was kept unchanged. Consistently, in glaucomatous patients, increased TNF-α concentration in aqueous humor and TNFR1 expression in the retina were observed, but TNFR2 expression levels showed unchanged [50]. However, it is noteworthy that activation of TNFR2 expressed in Müller cells promoted the production of TNF-α and may cause positive feedback to aggravate neuroinflammation in glaucoma [43, 59]. Therefore, the effects of TNFR2 in glaucoma remain to be addressed in the future. Furthermore, pre-injection of R7050 blocked the TNF-α-induced increase in the number of TUNEL-positive RGCs and partially reduced the number of apoptotic RGCs induced by IOP elevation, suggesting that TNF-α/TNFR1 signaling was indeed involved in RGC hyperexcitability and RGC injury in experimental glaucoma. These results are consistent with previous reports that neuronal hyperexcitability is associated with cell apoptosis [29–31, 60]. It should be noted that TNF-α-induced RGC hyperexcitability could increase Ca^2+^ influx through voltage-gated Ca^2+^ channels, together with activated Ca^2+^-permeable GluA2-lacking AMPA receptors [24], resulting in intracellular Ca^2+^ overload and triggering cellular death signaling [26].

### TNF-α induces RGC hyperexcitability by selectively upregulating Nav1.6 currents

VGSCs are essential for AP generation and conduction. Modulation of Na^+^ channels may change neuronal excitability [61, 62]. We found that TNF-α-induced RGC hyperexcitability was mediated by upregulating Na^+^ currents, as evidenced by the following facts. Firstly, intravitreal injection of TNF-α significantly and voltage-dependently increased Na^+^ current density in RGCs. Secondly, external application of TNF-α reversibly increased Na^+^ currents. Thirdly, TNF-α shifted the activation curves of Na^+^ currents toward hyperpolarizing direction, suggesting that TNF-α resulted in Na^+^ channel activation easier. These results are consistent with observations in dorsal root ganglion (DRG) neurons that TNF-α promoted neuronal excitability by increasing sodium channel currents densities, thus contributing to neuropathic pain hypersensitivity [42, 63–65].

One of the major findings in this study is that TNF-α induced RGC hyperexcitability was mediated by selectively upregulating Nav1.6 currents although RGCs express the other subtypes of Na^+^ channels, such as Nav1.1 and Nav1.2 [45–47]. Nav1.6 channel is one of the important subtypes of Na^+^ channels that determine neuronal excitability in the CNS [46, 66]. Even though the TTX-resistant Nav1.8 subtype of Na^+^ channels was found expressed in mouse RGCs [67, 68], our previous study has shown that in rat RGCs, Nav1.6 is a predominate subtype of Na^+^ channels, while TTX-resistant Na^+^ channels seem unlikely expressed in rat RGCs [47]. In the present study, we showed that TNF-α regulated Nav1.6 channels through two pathways. The first one is that TNF-α directly upregulated the currents through intracellular p38 MAPK and STAT3 signaling pathways [43, 44]. It should be noted that p38 MAPK and STAT3 are two important transcriptional regulation signaling molecules. They may transcriptionally activate Na1.6 expression, thus enhancing Nav1.6 currents. However, we found that blocking p38 MAPK and STAT3 signaling pathways completely eliminated the acute TNF-α application-induced enhancement of Na^+^ currents, but did not influence the increased Nav1.6 protein expression due to longer TNF-α exposure (7 days), suggesting that these two signaling pathways may directly modulate Na^+^ channels in a transcriptional-independent manner. Indeed, previous studies reported that p38 MAPK was involved in the upregulation of different subtypes of Na^+^ currents [42, 69–71], and activation of STAT3 directly increased the activity of electrogenic Na^+^/HCO_3_^-^ cotransporter 1 [72]. However, it was also reported that activation of p38 MAPK reduced the amplitude of peak Nav1.6 currents in the DRG-derived cell line ND7/23 and hippocampal neurons [71, 73]. It is possible that p38 MAPK may mediate different effects in different cells. The detailed mechanisms remain to be elucidated in our future study. The second one is that TNF-α upregulated the Nav1.6 expression in RGCs through NF-ĸB. NF-κB is one of the most important transcriptional factors. TNF-α has been reported to upregulate the expression of subtypes of Na^+^ channels, such as Nav1.7 and Nav1.3 [74, 75]. It is interesting that STAT3, another transcriptional factor, could promote the transcription and expression of Nav1.7-1.9 in DRG neurons [76], and TNF-α-activated STAT3 also facilitated Nav1.6 expression by increasing the histone H4 acetylation in *Scn8a* promoter in DRGs [64]. However, our data showed that inhibition of STAT3 failed to block the TNF-α-induced upregulation of Nav1.6 expression. Instead, STAT3 mediated the direct increase of Na^+^ currents by TNF-α in acutely isolated RGCs. In addition, immunohistochemical experiments showed that TNF-α-induced increase in Nav1.6 expression was mainly found in the axon-like structures. It was reported that Nav1.6 expressed in the distal of the AIS of cortical pyramidal neurons promoted action potential initiation [77]. Therefore, we speculated that upregulated expression of Nav1.6 in the TNF-α-injected retinas may contribute to RGCs hyperexcitability.

### Nav1.6 is a potential therapeutic target in glaucoma

Neuroinflammation plays an important role in the pathogenesis of multiple neurodegenerative diseases, including glaucoma, Alzheimer’s disease (AD), and Parkinson’s disease (PD) [78–81]. Nav1.6 is highly expressed in the nodes of Ranvier and AIS of neurons [47, 82], and neurons that expressed Nav1.6 show a higher excitability [83]. Abnormal Nav1.6 expression is closely related to various neuronal diseases [84–86]. In this study, we showed that TNF-α-mediated Nav1.6 upregulation was a major factor for RGC hyperexcitability and injury in glaucoma, suggesting that sodium channel blockers selectively targeting on Nav1.6 may be a potential therapeutic strategy in the treatment of glaucoma. Indeed, specific inhibition of Nav1.6 has showed to effectively relieve neuronal hyperexcitability and neuropathic pain [87, 88]. NBI 921352 (known as XEN 901), a novel small molecule Nav1.6 inhibitor, has been approved by FDA to initiate the phase 2 clinical trial in epilepsy patients [89].

## Conclusions

Taken together, all these results demonstrated that proinflammatory cytokine TNF-α induced the hyperexcitability of RGCs by enhancing Nav1.6 currents and protein expression through activating TNFR1, thus contributing to RGC apoptosis in glaucoma. Our results revealed a novel mechanism of TNF-α in glaucoma and provided a promising therapeutic target.

## Data Availability

All data generated or analyzed during this study are included in this published article.

## Abbreviations

AHTTX: 4,9-Anhydrotetrodotoxin
AP: Action potential
ACSF: Artificial cerebral spinal fluid
BCA: Bicinchoninic acid
COH: Chronic ocular hypertension
DRG: Dorsal root ganglion
d-APV: d-(-)-2-amino-5-phosphonopentanoic acid
IOP: Intraocular pressure
GCL: Ganglion cell layer
INL: Inner nuclear layer
IPL: Inner plexiform layer
OPL: Outer plexiform layer
ONL: Outer nuclear layer
R7050: 8-Chloro-4-(phenylthio)-1-(trifluoromethyl)-[1,2,4]triazolo[4,3-a]quinoxaline
RGCs: Retinal ganglion cells
TNF-α: Tumor necrosis factor-alpha
TNFR1: TNF-α receptor 1
TNFR2: TNF-α receptor 2
TUNEL: Terminal deoxynucleotidyl transferase dUTP nick end labeling
VGSCs: Voltage-gated sodium channels
